# Blood Gas Disturbances and Disproportionate Body Weight Distribution in Broilers With Wooden Breast

**DOI:** 10.3389/fphys.2020.00304

**Published:** 2020-04-07

**Authors:** Juniper A. Lake, Erin M. Brannick, Michael B. Papah, Cory Lousenberg, Sandra G. Velleman, Behnam Abasht

**Affiliations:** ^1^Center for Bioinformatics and Computational Biology, University of Delaware, Newark, DE, United States; ^2^Department of Animal and Food Sciences, University of Delaware, Newark, DE, United States; ^3^Department of Animal Sciences, Ohio Agricultural Research and Development Center, The Ohio State University, Wooster, OH, United States

**Keywords:** wooden breast, white striping, broiler, pectoralis major muscle, myopathy, pulmonary phlebitis

## Abstract

Wooden breast syndrome is a widespread and economically important myopathy and vasculopathy of fast growing, commercial broiler chickens, primarily affecting birds with high feed efficiency and large breast muscle yield. To investigate potential systemic physiological differences between birds affected and unaffected by wooden breast, a total of 103 market-age Cobb 500 broilers were sampled for 13 blood parameters and the relative weights of the pectoralis major muscle, pectoralis minor muscle, external oblique muscle, wing, heart, lungs, liver, and spleen. Blood analysis was performed on samples taken from the brachial vein of live birds and revealed significant differences in venous blood gases between affected and unaffected chickens. Chickens with wooden breast exhibited significantly higher potassium (K^+^) and lower partial pressure of oxygen (pO_2_), oxygen saturation (sO2), and pH. Additionally, affected males had significantly higher partial pressure of carbon dioxide (pCO_2_) and total carbon dioxide (TCO_2_) than unaffected males. Wooden breast affected broilers also possessed a significantly heavier pectoralis major muscle and whole feathered wing compared to unaffected broilers. Blood gas disturbances characterized by high pCO_2_ and low pH are indicative of insufficient respiratory gas exchange, suggesting that wooden breast affected broilers have an elevated metabolic rate that may also be inadequately compensated due to cardiovascular deficiencies such as poor venous return or respiratory insufficiency. Lung tissues from 12 birds with extreme sO_2_ values were subsequently examined to assess whether lung pathology contributed to the observed blood gas disturbance. Comparison of lung morphology between affected and unaffected birds revealed no apparent differences that could contribute to decreased parabronchial gas exchange. However, an interesting finding was the detection of pulmonary phlebitis in one of the wooden breast-affected samples consistent with vascular changes observed in pectoralis major muscle exhibiting the wooden breast phenotype. Our results suggest that the effects of wooden breast are not limited to the pectoralis major muscle and further indicate the importance of research into metabolic changes associated with the myopathy.

## Introduction

Modern commercial broiler chickens have undergone intensive selection for production traits such as high muscle yield, rapid growth, and high feed efficiency to meet consumer demand for low-cost lean chicken meat, specifically chicken breast meat. Such breeding strategies have produced remarkable results, nearly halving the time for birds to reach market weight while simultaneously increasing the breast muscle weight by about two-thirds since the 1950s (Petracci et al., 2015). Unfortunately, modern commercial broilers also experience increased prevalence of breast muscle myopathies, some of which cause substantial economic losses due to negative effects on meat quality (Kuttappan et al., 2016).

One such myopathy, commonly called wooden breast (WB), causes the pectoralis major muscle to become grossly pale, enlarged, and palpably firm, with visible signs of inflammation such as petechial hemorrhages and tissue edema (Sihvo et al., 2014). These macroscopic manifestations of the disorder are accompanied by considerable degradation of meat quality (Mudalal et al., 2015; Chatterjee et al., 2016) such that moderately or severely affected breast muscle cannot be sold as prime breast muscle filets and is instead condemned or sold for lower revenue products. In addition, increased locomotor difficulties, decreased wing mobility, and higher rates of dorsal recumbency among affected birds (Papah et al., 2017; Norring et al., 2018; Gall et al., 2019) suggest that WB may also be detrimental to bird welfare.

Research on WB and related myopathies is focused largely on the pectoralis major muscle, with only minor attention paid to potential systemic disparities accompanying the condition. However, factors that predispose broilers to WB – growth rate, feed efficiency, and breast muscle yield – can broadly be categorized as relating to general metabolism and body form. To date, there has been no comprehensive comparison of muscle and organ weights beyond the pectoralis major muscle weight and abdominal fat percentage between WB affected and unaffected broilers. An analysis of body weight distribution in WB birds may aid in identifying systemic physiological predisposition to or pathophysiologic effects of the myopathy. Intensive selection for commercially valuable traits has been shown to underpin biological imbalances in meat-type chickens, such as insufficient cardiopulmonary capacity to accommodate sustained rapid growth, resulting in pulmonary hypertension syndrome, or ascites (Wideman and French, 2000). It is known that WB-affected broilers possess larger breast muscles relative to body weight (Mutryn et al., 2015b) with higher cross-sectional areas (Dalle Zotte et al., 2017), and lower abdominal fat as a percentage of body weight (Mutryn et al., 2015b), but other potential differences in body weight distribution have not been examined.

Microscopic characterizations of WB have also largely been limited to the pectoralis major muscle (Sihvo et al., 2014, 2017, 2018; Papah et al., 2017), although evidence of altered blood gas values (Livingston et al., 2019a, b) in affected birds may indicate systemic disturbances or inadequate respiratory gas exchange. Two studies have provided initial insight into differences in blood parameters between WB-affected and unaffected broilers (Livingston et al., 2019a, b). Livingston et al. (2019a) evaluated the venous blood of male broilers at 35 days of age and found that WB severity was significantly associated with reduced partial pressure of oxygen (pO_2_) and increased total carbon dioxide (TCO_2_), bicarbonate (HCO_3_^–^), and base excess (BE). The same blood analysis conducted at 42 days of age produced similar results with regard to blood gas changes in WB affected birds, with the additional finding that packed cell volume (PCV, hematocrit; Hct) was significantly associated with WB severity (Livingston et al., 2019a). Livingston et al. (2019b) subsequently reported higher potassium (K^+^) levels in affected birds in a study evaluating the venous blood of male broilers at 42 days of age.

Thus, the objective of the current study was to further the systemic characterization of WB myopathy by comparing blood parameters, body weight distribution, and lung histology between affected and unaffected broilers.

## Materials and Methods

### Ethics Statement

The University of Delaware Institutional Animal Care and Use Committee approved the animal protocol (48R-2015-0) followed for this scientific study. Euthanasia was performed by means of cervical dislocation, and all efforts were made to maximize bird welfare.

### Experimental Animals and Wooden Breast Disease Scoring

This experiment was conducted in chicken houses located at the University of Delaware under environmental conditions simulating a commercial setting. As part of a genome-wide association study, a total of 542 Cobb 500 broilers from the same breeding population were raised in 4 chicken houses and provided free access to feed and water. At 47 days of age, 103 birds were selected for blood analysis based on manual palpation of the breast muscle. These birds were selected to achieve an approximately equal number exhibiting no palpable breast muscle firmness (48 total; 26 male and 22 female) and severe palpable firmness (55 total; 39 male and 16 female). Blood analysis using i-STAT requires sampling of blood from live birds. Therefore, bird selection was performed using breast muscle palpation of live birds while all statistical analyses utilized more accurate scoring of WB based on gross evaluation of the pectoralis major muscle at necropsy, as described below.

Birds were euthanized by cervical dislocation at 52 or 53 days of age. During necropsy, the pectoralis major muscles were evaluated for gross lesions and palpable firmness associated with WB and each bird was assigned a WB score using a 0–4 scale; 0-Normal indicates the bird had no macroscopic signs of the myopathy, 1-Very Mild indicates 1% or less of the breast muscle was affected, 2-Mild indicates between 1% and 10% of the breast muscle was affected, 3-Moderate indicates between 10 and 50% of the breast muscle was affected, and a score of 4-Severe indicates that more than 50% was affected. This scoring system is the same one implemented by Lake et al. (2019) and separates unaffected and mildly and moderately affected chickens with higher resolution.

### Blood Analysis

At 47 days of age, 1 ml of blood was drawn from the brachial wing vein of each bird using a 3 ml syringe with 23-gauge needle that had been prepared by aspirating and expelling a small volume of liquid heparin prior to blood collection. The blood was deposited immediately into a new i-STAT CG8 + cartridge inserted in the i-STAT 1 Analyzer (model 300A, Abbott Point of Care Inc., Princeton, NJ, United States) to perform rapid blood analysis. While designed for clinical use in humans, the i-STAT system’s performance in *Gallus gallus* has been demonstrated in previous studies (Steinmetz et al., 2007; Livingston et al., 2019a). CG8 + test cartridges were used to test blood chemistry parameters including sodium (Na^+^), K^+^, ionized calcium (iCa), and glucose (Glu); hematologic parameters Hct and hemoglobin (Hb); and blood gas parameters including pH, partial pressure of carbon dioxide (pCO_2_), TCO_2_, HCO_3_^–^, BE, oxygen saturation (sO_2_), and pO_2_. After all measurements were completed, data was downloaded from the analyzer and consolidated for statistical analysis. Body weight at 47 days was also measured at this time.

### Body Weight Distribution

The same 103 birds used for blood analyses were also used to evaluate body weight distribution. After euthanasia, the left pectoralis major muscle, left pectoralis minor muscle, left external oblique muscle, heart, lungs, liver, spleen, and whole feathered left wing, disarticulated at the shoulder, were dissected from each bird. The weight of each dissected body part was recorded, along with the body weight of the bird before necropsy.

### Statistical Analysis

Statistical analyses were performed using JMP software (SAS Institute, Cary, NC, United States). To improve statistical power, birds were grouped according to WB status: unaffected birds included those assigned a WB score of 0-Normal or 1-Very Mild and affected birds included those assigned a score of 2-Mild, 3-Moderate, or 4-Severe. All i-STAT measurements were analyzed using a mixed linear model with WB status, sex, WB-sex interaction, and body weight at 47 days as fixed effects and poultry house as a random effect. Body part weights were analyzed using a mixed linear model with WB status, sex, WB-sex interaction, and body weight at necropsy as fixed effects and poultry house as a random effect. Effects with *P* ≤ 0.05 were considered significant for all tests.

### Histological Evaluation of Lungs

Of the 103 broilers used in this study, six birds (3 males and 3 females) with the lowest sO_2_ values and six birds with the highest sO_2_ values (3 males and 3 females) were selected for microscopic examination of lung tissue based on blood gas values measured at 47 days of age. In the low sO_2_ group, 5 birds were classified as affected, with WB scores of 3-Moderate or 4-Severe, and 1 bird was classified as unaffected, with a WB score of 0-Normal. In the high sO_2_ group, all 6 birds were classified as unaffected, with a WB score of 0-Normal. From each of the selected birds, lung tissue from the cranial and caudal aspects from either the left or right lung were harvested and fixed by immersion in 10% neutral buffered formalin. Samples were processed routinely for staining with hematoxylin and eosin as described by Papah et al. (2017) before histologic evaluation with a light microscope and morphometric analysis with the Aperio LV1 digital microscope (Leica Biosystems, Buffalo Grove, IL, United States).

Lung tissue was examined microscopically by a veterinarian (Papah) and a certified veterinary anatomic pathologist (Brannick) for histopathologic lesions or other tissue changes which could affect systemic blood parameters, such as the presence of inflammation, fibrosis, lymphoid follicular hyperplasia (lymphocytic nodules), cartilaginous nodules, edema or hemorrhage in the gas exchange areas, thickening of parabronchial walls, and obstruction of parabronchi. Assessment of all slides was performed in a blinded fashion and later microscopic lesions were assessed for an association with sO_2_ status (high or low), sex, or WB status.

## Results and Discussion

### Body Weight Distribution

The effects of WB, sex, WB-sex interaction, and body weight on the weights of the dissected left pectoralis major muscle, left pectoralis minor muscle, left external oblique muscle, whole feathered left wing, heart, lungs, liver, and spleen are provided in Table 1. Wooden breast had a significant association with the pectoralis major muscle and whole feathered left wing, which were both larger in affected birds compared to unaffected birds. Previous studies have found similar results with regard to pectoralis major yield (Mutryn et al., 2015b; Livingston et al., 2019b), providing fodder for speculation that high breast muscle yield is responsible for development of the WB and WS phenotypes due to overstretching of the myofibers and a reduction in capillary density (Kuttappan et al., 2013; Dalle Zotte et al., 2017). However, microscopic lesions of WB can be detected as early as 1 week of age in the pectoralis major muscle (Papah et al., 2017). Thus, hypotheses suggesting that WB arises from overstretching and ischemia may be inadequate or incomplete. Without discounting the potential contribution of pectoralis major growth rate to WB development, it is important to consider alternative interpretations of these results. For example, it has been proposed that muscle hypertrophy may be symptomatic of WB rather than causal, similar to the pathological hypertrophy of organs in chronic complications of diabetes mellitus in mammals (Lake et al., 2019; Lake and Abasht, 2020).

**TABLE 1:** Effects of wooden breast (WB) status, sex, the interaction of WB and sex, and body weight on the weight of the left pectoralis major, left pectoralis minor, left whole feathered wing, left external oblique, heart, lungs, liver, and spleen of broiler chickens.

		WB			Sex			WB × Sex					Body Weight
					
								Affected	Affected	Unaffected	Unaffected		
		Affected	Unaffected	*P*-value	Male	Female	*P*-value	Male	Female	Male	Female	*P*-value	*P*-value
Broilers (n)		55	48		65	38		39	16	26	22		
P.major (g)	Mean	425.51	400.59	0.002	402.70	423.41	0.079	417.84	433.19	387.56	413.63	0.505	<0.001
	SE	4.67	4.68		4.54	7.71		6.66	10.42	6.78	9.41		
P.minor (g)	Mean	81.66	83.58	0.305	80.26	84.97	0.074	81.14^a,b^	82.17^a,b^	79.38^a^	87.78^b^	0.047	<0.001
	SE	1.55	1.61		1.54	2.06		1.89	2.61	1.98	2.41		
Wing (g)	Mean	169.62	160.31	<0.001	169.07	160.87	0.008	174.72	164.53	163.42	157.20	0.350	<0.001
	SE	1.78	1.85		1.77	2.38		2.18	3.01	2.28	2.78		
Ext. obl. (g)	Mean	7.53	7.03	0.213	6.88	7.68	0.153	7.60^a^	7.46^a,b^	6.15^b^	7.90^a^	0.018	0.006
	SE	0.26	0.28		0.26	0.40		0.35	0.52	0.37	0.48		
Heart (g)	Mean	17.74	17.83	0.867	17.84	17.74	0.893	17.86	17.62	17.81	17.85	0.786	<0.001
	SE	0.32	0.33		0.31	0.50		0.44	0.67	0.46	0.61		
Lungs (g)	Mean	15.51	16.35	0.209	17.23	14.64	0.006	16.58	14.45	17.88	14.83	0.483	0.158
	SE	0.52	0.54		0.52	0.71		0.65	0.91	0.68	0.84		
Liver (g)	Mean	62.43	64.19	0.334	60.09	66.53	0.013	58.17	66.70	62.02	66.35	0.241	<0.001
	SE	1.30	1.36		1.29	1.86		1.68	2.42	1.76	2.22		
Spleen (g)	Mean	4.20	4.46	0.223	4.03	4.62	0.056	3.83	4.57	4.24	4.67	0.460	<0.001
	SE	0.13	0.14		0.14	0.21		0.19	0.27	0.20	0.25		

*Analysis was performed using a mixed linear model approach with poultry house included as a random effect. Data are presented as the least square mean and standard error. Means not sharing a common superscript letter within the interaction effect are significantly different (*P* < 0.05, Tukey’s HSD test).*

The external oblique muscle showed an interesting effect of the WB-sex interaction, with affected males and unaffected females having the highest average weights of those muscles and unaffected males having the lowest weights. Similarly, the weight of the pectoralis minor muscle was highest in unaffected females and lowest in unaffected males. One potential explanation for the observed group means of the external oblique muscle is as an adaptive response to the size of the combined pectoralis major and pectoralis minor muscle. Avian respiration relies on movement of the sternum to allow expansion of the bellows-like air sacs during inhalation (Schmidt-Nielsen, 1971). The external obliques are ventilatory muscles that insert onto the base of the uncinate processes of the ribs, extensions of bone that project caudally from the vertical segment of each rib, and move the sternum dorsally during expiration (Codd, 2005). Because inhalation and exhalation are active processes driven by musculoskeletal movements, additional weight, especially on the sternum increases the metabolic demand of respiration, reduces the overall effectiveness of respiratory movements (Tickle et al., 2014), and may result in strengthening of respiratory muscles. This is in accordance with unaffected males having the smallest pectoralis major muscle, pectoralis minor muscle, and external oblique muscle in the present model.

Compared to females in the present study, males possessed larger wings and lungs, but a smaller liver after accounting for WB and body weight. There was no significant difference in the size of the pectoralis major muscle between males and females in our model. Body weight had a significant effect on the sizes of all body parts except the lungs, with larger birds possessing generally heavier body parts but relatively smaller lungs.

### Blood Analysis

The effects of WB, sex, WB-sex interaction, and body weight on 13 blood parameters are shown in Table 2. Compared to unaffected birds, WB affected birds exhibited significantly higher venous K^+^ and significantly lower pH, sO_2_, and pO_2_. Affected male birds also possessed significantly higher pCO_2_ and TCO_2_ values compared to unaffected male birds. Although affected female birds had pCO_2_ and TCO_2_ values higher than those of unaffected female birds, the effect was not significant potentially due to the smaller number of female birds sampled. These results are largely in accordance with previously published data of blood parameters measured at 42 days of age (Livingston et al., 2019a, b), although no significant effect of WB on Hct or BE was detected in the present study. The present model also detected a significant sex effect for BE and a WB-sex interaction effect for Glu, with males possessing higher BE than females and affected males possessing higher Glu compared to affected females. It is unclear what might be causing these specific sex effects.

**TABLE 2:** Effects of wooden breast (WB) status, sex, the interaction of WB and sex, and body weight on blood sodium (Na^+^), potassium (K^+^), ionized calcium (iCa), glucose (Glu), hematocrit (Hct), hemoglobin (Hb), pH, partial pressure of carbon dioxide (pCO_2_), total carbon dioxide (TCO_2_), partial pressure of oxygen (pO_2_), oxygen saturation (sO_2_), bicarbonate (HCO_3_^–^), and base excess (BE).

		WB			Sex			WB x Sex					Body Weight
					
								Affected	Affected	Unaffected	Unaffected		
		Affected	Unaffected	*P*-value	Male	Female	*P*-value	Male	Female	Male	Female	*P*-value	*P*-value
Broilers (n)		55	48		65	38		39	16	26	22		
Na^+^ (mmol/L)	Mean	150.17	149.24	0.123	149.91	149.50	0.611	150.05	150.30	149.78	148.71	0.240	0.320
	SE	0.42	0.47		0.42	0.60		0.58	0.75	0.56	0.74		
K^+^ (mmol/L)	Mean	5.04	4.86	0.045	4.98	4.91	0.569	5.11	4.97	4.85	4.86	0.372	0.127
	SE	0.05	0.06		0.05	0.09		0.08	0.11	0.08	0.11		
iCa (mmol/L)	Mean	1.41	1.39	0.416	1.40	1.40	0.983	1.41	1.41	1.40	1.39	0.932	0.266
	SE	0.02	0.02		0.02	0.02		0.02	0.02	0.02	0.02		
Glu (mmol/L)	Mean	220.06	222.58	0.366	224.29	218.36	0.119	226.71^a^	213.42^b^	221.87^a,b^	223.30^a,b^	0.006	0.310
	SE	1.85	2.08		1.87	2.75		2.61	3.43	2.52	3.39		
Hct (%PCV)	Mean	23.79	22.84	0.151	23.31	23.32	0.994	23.82	23.76	22.80	22.87	0.919	0.216
	SE	0.50	0.55		0.50	0.69		0.67	0.85	0.64	0.83		
Hb (g/dL)	Mean	8.08	7.76	0.161	7.93	7.92	0.989	8.10	8.07	7.75	7.78	0.903	0.211
	SE	0.17	0.19		0.17	0.24		0.23	0.29	0.22	0.29		
pH	Mean	7.360	7.389	0.007	7.382	7.367	0.268	7.364	7.356	7.401	7.377	0.396	0.600
	SE	0.008	0.009		0.008	0.011		0.011	0.014	0.010	0.013		
pCO_2_ (mmHg)	Mean	47.26	42.16	0.002	44.69	44.73	0.984	48.82^a^	45.70^a,b^	40.55^b^	43.77^a,b^	0.041	0.825
	SE	0.88	1.02		0.86	1.55		1.42	1.95	1.26	1.96		
TCO_2_ (mmol/L)	Mean	27.81	26.87	0.125	28.01	26.67	0.111	29.06^a^	26.57^a,b^	26.96^b^	26.78^a,b^	0.045	0.333
	SE	0.38	0.43		0.38	0.58		0.55	0.74	0.53	0.73		
pO_2_ (mmHg)	Mean	39.85	44.75	<0.001	42.03	42.57	0.751	39.52	40.18	44.54	44.97	0.923	0.352
	SE	1.16	1.22		1.15	1.48		1.42	1.79	1.38	1.71		
sO_2_ (%)	Mean	70.57	79.07	<0.001	74.86	74.78	0.974	70.20	70.94	79.52	78.62	0.647	0.452
	SE	1.37	1.49		1.35	1.95		1.83	2.46	1.76	2.34		
HCO_3_^–^ (mmol/L)	Mean	26.42	25.55	0.133	26.66	25.31	0.087	27.62	25.22	25.70	25.40	0.052	0.371
	SE	0.36	0.41		0.36	0.55		0.53	0.70	0.50	0.69		
BE (mmol/L)	Mean	1.00	0.46	0.375	1.60	–0.13	0.036	2.32	–0.31	0.88	0.05	0.113	0.443
	SE	0.42	0.47		1.60	0.61		0.58	0.75	0.56	0.74		

*Analysis was performed using a mixed linear model approach with poultry house included as a random effect. Data are presented as the least square mean and standard error. Means not sharing a common superscript letter within the interaction effect are significantly different (*P* < 0.05, Tukey’s HSD test).*

Elevated pCO_2_ in conjunction with a decline in pH in WB affected birds is possibly indicative of an acid-base disorder called respiratory acidosis. While respiratory acidosis is classically defined in terms of arterial blood gas measurements, the high correlation of arterial and venous pCO_2_ and pH is well-established in humans, dogs, and chickens, as is the use of venous measurements for investigation of acid-base disturbances (Forster et al., 1972; Ilkiw et al., 1991; Wideman et al., 2003; Yildizdaş et al., 2004). Respiratory acidosis occurs when the body produces more CO_2_ than can be removed by the lungs (D’Addesio, 1992). As carbon dioxide accumulates in the blood, it causes blood pH to decrease, triggering compensatory mechanisms in the kidneys, such as HCO_3_^–^ retention, to mitigate the rising acidity. Elevated HCO_3_^–^ levels are suggestive of renal compensation and possible chronic respiratory acidosis, as the kidneys increase excretion of acid and hydrogen ions and increase reabsorption of HCO_3_^–^ (Carter et al., 1959). Livingston et al. (2019a) previously found increased HCO_3_^–^ levels in WB-affected broilers. In the present study, HCO_3_^–^ values are higher in affected males compared to unaffected males, though the WB-sex interaction effect is not quite significant in our model (*P*-value = 0.052).

Respiratory acidosis is caused either by an increase in CO_2_ production (increased metabolism), a relative decrease in respiratory gas exchange (cardiopulmonary insufficiency), or both (Epstein and Singh, 2001). Previous studies comparing feed-restricted vs. non-feed-restricted broilers and slow-growing vs. fast-growing broilers (Julian and Mirsalimi, 1992; Olkowski et al., 1999) have demonstrated how an increase in metabolism can cause changes to blood gas values similar to those seen in the present study. Similarly, respiratory acidosis caused by cardiopulmonary insufficiency has been demonstrated by comparing ascitic versus non-ascitic chickens (Malan et al., 2003). In the present study, body weight was investigated for a potential association with blood parameter values but did not show any significant effects (Table 2). The weight of the whole feathered left wing was also tested as a main effect for blood gas values as the brachial vein, from which blood samples were taken, drains the peripheral wing tissue. However, it also showed no significant effects for blood gas values and so was excluded (data not shown). The fact that WB has a significant effect on blood gas values beyond what can be explained by body weight or wing weight suggests that a higher metabolic rate due to faster growth is not the cause of the apparent blood gas disturbance. It is, therefore, important to explore other potential metabolic, respiratory, or cardiovascular differences between WB affected and unaffected birds that might explain the results seen in this study and in previous studies (Livingston et al., 2019a, b).

Metabolically, WB involves substantial alterations in the pectoralis major muscle, including an apparent increase in lipid metabolism and decrease in glycolysis (Mutryn et al., 2015a; Abasht et al., 2016; Papah et al., 2018). Increased reliance on lipids for energy production rather than glucose can raise oxygen consumption due to the lower phosphate/oxygen ratio of fatty acids, but would not be expected to raise CO_2_ production (Brand, 2005). Other features of WB, such as hypercontraction of muscle fibers (Velleman et al., 2018) or increased activity of ATP-powered calcium pumps, could raise the body’s energy requirements and total metabolic rate without contributing to growth rate. Sarcoplasmic/endoplasmic reticulum Ca^2+^-ATPase (SERCA) pumps have been found to account for 40–50% of the resting metabolic rate in mouse skeletal muscle, or 12–15% of whole body resting oxygen consumption (Smith et al., 2013). Upregulation of SERCAs in the pectoralis major muscle of affected birds at market age is supported by transcriptional and proteomic evidence (Mutryn et al., 2015a; Soglia et al., 2016), and the disruption of intracellular calcium homeostasis has been identified as a key feature of the early pathogenesis of WB (Papah et al., 2018; Lake et al., 2019). The contribution of sarcoplasmic reticulum calcium cycling to increased resting energy expenditure in the WB phenotype has been proposed (Lake and Abasht, 2020) but remains unexplored experimentally.

While numerous potential pulmonary causes of insufficient gas exchange exist, none are particularly well-supported by existing knowledge of WB or the results of the present study. Our data indicate WB is not associated with reduced lung size, altered lung morphology (see following section), or reduced respiratory musculature (i.e., the external oblique muscle).

However, the circulatory system is also intimately involved in respiratory exchange and can reduce gas exchange at the blood-gas barrier by decreasing the rate at which blood passes by the gas exchange surface in the lungs (Ludders, 2015). Evaluations of microscopic lesions associated with WB have cataloged extensive damage to the veins in the p. major muscle of affected birds as well as evidence of hemodynamic perturbations resulting from such damage (Papah et al., 2017; Sihvo et al., 2017). In many cases, venous inflammation progresses to circumferential transmural and valvular infiltration of veins by inflammatory cells (Papah et al., 2017). Subsequent congestion, edema, valvular damage, and stenosis (narrowing) or obstruction of the venous lumen (Papah et al., 2017; Sihvo et al., 2017) all indicate significant impairment of venous return and, implicitly, a reduction of cardiac output. Poor venous return in affected birds could lead to increased accumulation of metabolic waste including CO_2_, as indicated by blood gas analysis in this study, and negatively affect gas exchange by slowing the flow of blood back to the gas exchange surfaces of the lungs. Impaired venous return from phlebitis may exacerbate what some characterize as existing vascular insufficiency in the pectoralis major of commercial broilers. At the cellular level, large breast muscles are produced by increasing the number, diameter, and length of muscle fibers (Scheuermann et al., 2004; Roy et al., 2006), causing a reduction of capillary density among other effects (Hoving-Bolink et al., 2000; Joiner et al., 2014).

Apart from blood gases, the only other analyte measurement that was significantly altered in affected birds in the present study was potassium. Hyperkalemia, increased K^+^, is a known effect of high blood CO_2_ and therefore a common symptom of respiratory acidosis (Scribner et al., 1955; Ladé and Brown, 1963; Kilburn, 1966). Extracellular K^+^ concentration is tightly regulated in the body to maintain it within the necessary range for cellular functions such as electrical excitability of cardiac and skeletal muscle (Aronson and Giebisch, 2011). The majority of the body’s K^+^ is located in the intracellular fluid of skeletal muscle and is shifted between muscle cells and extracellular space by the activity of various ion transport pathways (Youn and McDonough, 2009). A net loss of K + from cells during respiratory acidosis is mediated primarily by Na^+^-H^+^ exchange and Na^+^/K^+^-ATPase activity, although extracellular elevation of bicarbonate enhances Na^+^-HCO_3_^–^ cotransport and prevents the severity of hyperkalemia seen in metabolic acidosis where bicarbonate levels are reduced (Aronson and Giebisch, 2011).

### Histological Evaluation of the Lungs

**FIGURE 1:**
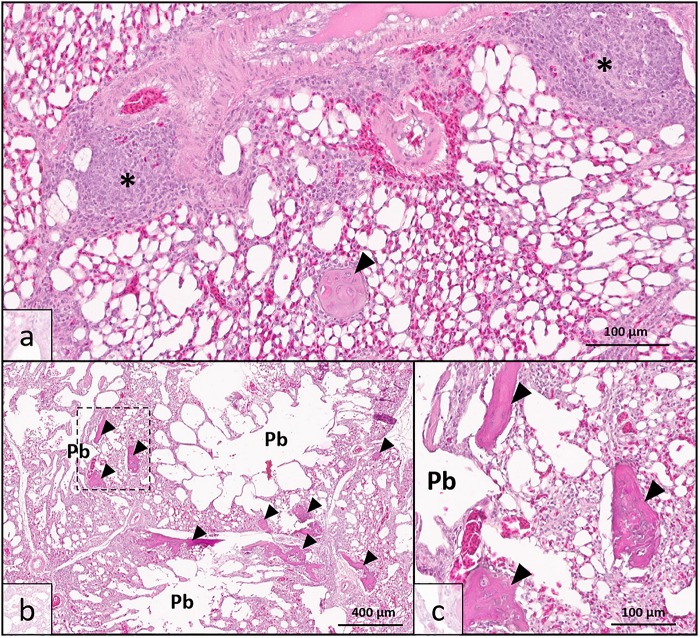
Lung histopathology of wooden breast-affected broiler chickens with low venous blood oxygen saturation (sO_2_). **(a)** Obliterative lymphoplasmacytic venous inflammation, interpreted as phlebitis (*), was observed in one bird with sparing of adjacent arterial vessel (top) consistent with the wooden breast phenotype in muscle tissues. Chondro-osseous metaplasia (arrowhead) may be occurring as a sequela of low oxygenation in pulmonary tissues. **(b)** Multiple parabronchi (Pb) exhibit chondro-osseous metaplasia (arrowheads) in wooden breast-affected birds with low sO_2_ values. The number and size of metaplastic foci were greater than either wooden breast-affected birds with high sO_2_ values or unaffected birds. **(c)** Higher magnification of region demarcated by dotted line in 1b showing metaplastic foci in greater detail. [Hematoxylin and Eosin stain, scale bar indicates 100 μm in **(a,c)** and 400 μm in **(b)**].

Histologic analysis demonstrated that there was no clinically significant lung disease (pneumonia, etc.) observed in tissues from either high sO_2_ or low sO_2_ birds to explain detectable differences in blood oxygen saturation. A single lung specimen from the low sO_2_ group exhibited moderate localized multifocal lymphoplasmacytic phlebitis consistent with vascular changes observed in WB musculature (Figure 1a) and was confirmed to have been collected from a low sO_2_ WB affected bird following analysis. All pulmonary tissues examined from both high sO_2_ and low sO_2_ birds exhibited one or more foci of chondro-osseous metaplasia, characterized by focal to multifocal islands of well-differentiated non-neoplastic cartilage or bone tissue within otherwise healthy pulmonary tissue. Chondro-osseous metaplasia is thought to arise from within pulmonary connective tissue over time in response to low oxygenation in tissues (chronic hypoxia) and has been previously reported in broiler chickens in association with ascites syndrome (Maxwell, 1988). In the present study, there was no gross evidence of fulminant ascites in any of the birds examined. While present in both WB affected and WB unaffected birds, the metaplastic change in pulmonary tissues was more extensive resulting in larger, more numerous metaplastic foci in WB affected birds (Figures 1b,c). This finding may indicate that while lung tissues are susceptible to hypoxic injury even in clinically normal broilers, the WB condition may exacerbate tissue changes due to regionalized or systemic hypoxia.

Other minor histopathologic findings included bronchus-associated lymphoid tissue (BALT) hyperplasia, occasionally with discrete nodular follicle formation, and extramedullary hematopoiesis (EMH). Generalized and nodular lymphoid hyperplasia likely indicate immune response to inhaled antigens or irritants and are not suspected to be related to WB. The airway associated lymphoid tissues did not appear to impinge upon or obstruct airway lumens. The physiologic process of EMH is common in tissues of young animals, but can sometimes occur to produce additional red blood cells in response to low tissue oxygenation. However, the limited extent and level of EMH in the lung tissues in the present study indicate that EMH is likely an incidental rather than clinically significant finding. Mild, limited EMH is a common finding in avian tissues, including lung, even in clinically normal birds. Artifactual changes from specimen collection and processing, such as acute hemorrhage with no tissue reaction and collapse of air spaces with no indication of true airway obstruction (atelectasis) were observed but disregarded for purposes of analysis.

Altogether, WB status, sex, or sO_2_ status were not associated with histopathologic changes in the lungs, suggesting that structural abnormalities or disease in the lungs are not likely contributors to the WB phenotype. The chondro-osseous metaplasia lesions observed in this study are likely sequelae of the WB syndrome rather than precipitating factors in the development of the disease. The finding of pulmonary phlebitis in one WB bird suggests that the phlebitis associated with WB is not restricted to the musculature but may be systemic in nature.

Previously, our laboratory reported altered gene expression in lung tissue of WB affected broilers (Wong, 2018) which may be connected with the present finding of pulmonary phlebitis. In that study, the top significant canonical pathways identified from the list of differentially expressed genes included atherosclerosis signaling, adipogenesis, and LXR/RXR activation, a pathway involved in the regulation of lipid metabolism and inflammation. Increased expression of genes involved in lipid metabolism and uptake, such as *lipoprotein lipase* (*LPL*), *fatty acid binding protein 4* (*FABP4*), and *adiponectin c1q and collagen domain containing* (*ADIPOQ*), in the lung tissue of WB affected broilers (Wong, 2018) is reminiscent of the transcriptomic changes associated with WB in the pectoralis major muscle (Papah et al., 2018; Lake et al., 2019) and may reflect a similar mechanism. Papah and Abasht (2019) recently found increased *LPL* expression in veins undergoing phlebitis in the pectoralis major muscle of WB affected birds, further substantiating the link between increased lipid metabolism and venous inflammation. In light of this knowledge, it is possible that phlebitis in the lungs is more widespread and not fully represented by the limited number of sections and samples examined in the present study.

## Conclusion

The findings of the present study indicate that WB is associated with blood gas disturbances characterized primarily by increased venous K^+^ and pCO_2_ and decreased pH, sO_2_ and pO_2_. The accumulation of carbon dioxide and acidification of venous blood occurs when the metabolic demands of the tissue exceed the capacity of the respiratory or circulatory system. Factors that may contribute to increased metabolic demand in WB affected birds include hypercontraction of muscle fibers and, importantly, the dysregulation of calcium homeostasis. Cardiovascular and pulmonary deficiencies, specifically venous damage caused by phlebitis and disproportionate growth of the pectoralis major compared to respiratory muscles, potentially also contribute to inadequate respiratory gas exchange in affected birds. Blood gas disturbances, musculature differences, and pulmonary chondro-osseous metaplasia and phlebitis demonstrated herein may further indicate broader systemic implications of the metabolic dysfunction and circulatory insufficiency already described in existing literature regarding WB. Since these results were obtained in 7-week-old birds, findings are more informative of the WB myopathy’s effects (sequelae) rather than its etiology and further studies are required to ascertain whether a blood gas disturbance is present during early stages of the disease.

## Data Availability Statement

The datasets generated for this study are available on request to the corresponding author.

## Ethics Statement

The animal study was reviewed and approved by the University of Delaware Institutional Animal Care and Use Committee.

## Author Contributions

BA conceptualized, designed, and supervised the study. JL performed the statistical analysis and wrote the original draft of the manuscript. EB, MP, and CL conducted histopathological examination of lung samples and EB wrote sections of the manuscript relevant to that examination. BA, JL, SV, EB, and MP contributed to manuscript revision. All authors read and approved the submitted version.

## Conflict of Interest

The authors declare that the research was conducted in the absence of any commercial or financial relationships that could be construed as a potential conflict of interest.
